# Taking evo-devo to the next level

**DOI:** 10.1073/pnas.2519801122

**Published:** 2025-10-06

**Authors:** Leslea J. Hlusko

**Affiliations:** ^a^Paleobiology Program, Centro Nacional de Investigación sobre la Evolución Humana, Burgos 09002, Spain

Mammals have been on Earth for more than 200 Myr, but it was not until the demise of dinosaurs 65 Mya that they hit their evolutionary stride, diversifying into a wide range of body sizes and successfully exploiting environments from Arctic ice to deep ocean waters and most of the terrestrial options in between (1, 2). A key innovation that facilitated this radiation was the evolution of heterodonty, a dentition with incisors, canines, premolars, and molars. These different tooth types are able to evolve somewhat independently, facilitating the appearance of an incredible range of dental patterns, tooth shapes, and sizes that enabled a broad array of dietary and behavioral specializations (3). A long-running research aim in evolutionary biology seeks to understand the developmental and molecular mechanisms that underlie mammalian heterodonty. Molecular innovations in developmental biology over the last few decades have been applied to this quest in earnest. Our initial insights from mouse developmental genetics were enthusiastically embraced as being generalizable to all mammals (4). However, biologists increasingly found that the mouse model is not as explanatory as initially assumed, even for some closely related taxa (e.g., refs. 5–7). In a recent publication in this journal, Lafuma et al. (8) address this disconnect with an elegant study of vole dentitions that offers insight as to how voles evolved their unusually complex first molar morphologies, combining evidence from vole development with the morphological variation observed across 82 living and 69 extinct species.

Voles prove to be a wonderful system for an interdisciplinary study of the evolution of molar complexity. During the Plio-Pleistocene they diversified across a wide range of environments throughout the Northern Hemisphere and are well preserved in the fossil record. Voles are evolutionarily close to mice and amenable to being raised in the laboratory. Therefore, they can easily be compared to mouse developmental studies. Coincidentally, vole molars differ from mice in having extreme levels of cuspal complexity. In their macroevolutionary study, Lafuma et al. (8) find that voles added cusps in a pair-wise fashion to a five-cusped ancestor, quickly and repeatedly reaching nine cusps. Over longer periods of time, some lineages achieved another pair, resulting in first molars with 11 cusps. They also found that cusp number correlates positively with the length of the molar. However, this phenomenon is not necessarily a result of crown elongation, as cusp number is negatively correlated with the width. Across the macroevolutionary scale, Lafuma et al. (8) observe that the number of cusps also increased when the length of the crown was stable but the width narrowed, suggesting that the developmental and genetic influences on molar length differ from those influencing molar width.

Lafuma et al. (8) test this hypothesis with a developmental approach. Using microCT of embryos, they were able to compare the earliest stages of molar development between voles and mice, at the time point when the earliest-developing cusp begins to appear. They found that the next 24 h of development revealed a distinct difference between the two taxa: Vole molars grow at a much accelerated pace compared to mice, leading to the development of additional cusp pairs. After this early burst of accelerated vole growth slows down, the mouse growth rate increases and surpasses it. But for mice, by this point in the developmental process there is less opportunity for modification of cusp patterning, restraining mice from achieving the high number of cusps seen in voles. This discovery demonstrates how important the timing of differential growth is for dental variation—the much earlier accelerated growth is the key mechanism for unlocking the extreme cuspal complexity characteristic of voles.

In PNAS, Lafuma et al. (8) address this disconnect with an elegant study of vole dentitions that offers insight as to how voles evolved their unusually complex first molar morphologies, combining evidence from vole development with the morphological variation observed across 82 living and 69 extinct species.

This type of comparative approach between development and macroevolutionary morphological variation is adding important insight to our understanding of how the mammalian dentition is formed. In another example, Sadier et al. (9) combined development with neontological variation across noctilionoid bats. Similar to the insights of Lafuma et al. (8), the diversity of bat dentitions is also achieved through the differential timing of growth rate shifts. However, in bats, the diversity of dental patterns appears to result from the interplay of jaw growth rates with two different activation/inhibition mechanisms that operate in opposite directions, one for molars and another for premolars (9). The interaction of the timing of shifts in jaw growth rate with premolar and molar cascades seems to be sufficient to achieve the large range of jaw length, tooth number, tooth size variation, and evolutionary convergence observed across these bats.

Interestingly, Lafuma et al. (8) also found that the development of the surrounding jaw influences vole molar shape. Their ex vivo experiments of molar growth supported their interpretation that a very early growth spurt is essential to achieving the complex occlusal morphology of voles. Additionally, they found that growing a tooth outside of the jaw also revealed that tooth proportions differ markedly when they do not have the in vivo conditions of the surrounding jaw bone: Ex vivo molars grow wider and have an even more diminished cuspal complexity than is observed across the fossil and extant morphological variation, aligning with their observation from the macroevolutionary evidence that molar length and width appear to reflect different biological influences.

Evolutionary biology in the 1990s and early 2000s was full of excitement over the new discipline of evo-devo made possible by technological advances in molecular biology, fully embracing the concept that genetic control of development is essential to the evolution of novelty (10, 11). However, for practical reasons this discipline largely focused on the comparison of a handful of extant model organisms for the evo component, though of course with exceptions (e.g., ref. 12). Mice were considered to be stand-ins for mammals generally, and fossils and macroevolutionary patterns of variation were mostly seen as useful tools for testing mechanistic models derived from mouse developmental studies (e.g., ref. 4).

The research of Lafuma et al. (8) and Sadier et al. (9) demonstrate that mammalian evo-devo is clearly advancing in two important directions. First, these projects use macroevolutionary information in conversation with developmental studies, letting each inform how we understand the other. Additionally, they broaden the taxonomic sampling, enabling comparisons with the well-established mouse model across taxonomic space, development process, and through geological time. These comparisons yield a more insightful understanding of mammalian tooth development, demonstrating how the diversity of mammalian dentitions results from tinkering at different time-points and with different mechanisms in a more complex way than could be inferred from just one species, especially one with such a reduced dentition as mice. The revelation of these differences (and similarities) is teaching us quite a bit about how mammalian dentitions develop generally, with implications for human health through regenerative dentistry (13).

In his description of evo-devo, Müller (11) credits Pere Alberch as a major inspiration for the discipline. A biologist who passed away much too young, Alberch advocated that development and genetics cannot be thought of as different levels of biology, but rather it is their integration, their evolvability that is essential to unlocking the mysteries of how evolution works (14). Evolvability—the concept of a taxonomic group’s ability to morphologically diversify, is quantified as a measure of the relationship between phenotypic and genetic variance within a population and is the substrate on which evolution operates (15–17). Given that evolvability is a population-level measure, in order to realize the original vision for evo-devo, quantitative genetics will need to be more integrated into our studies of development.

**Fig. 1.**
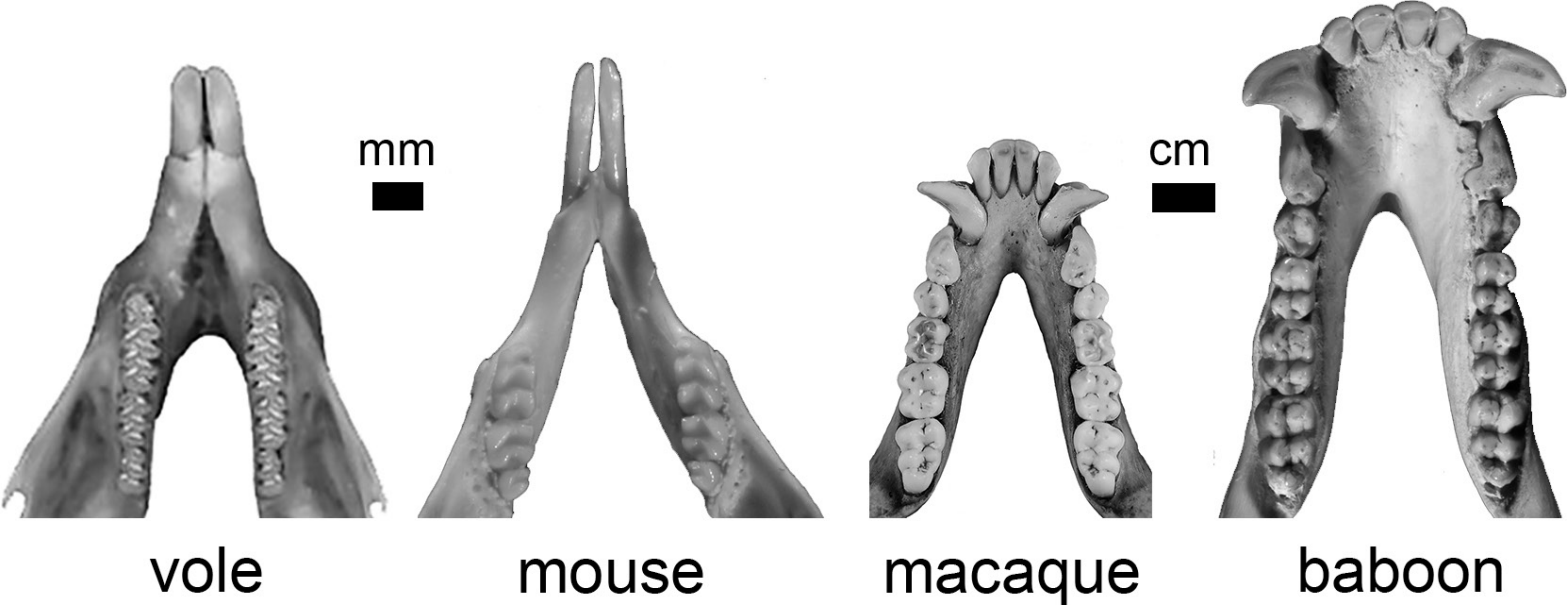
Comparison of the dental complexity of two rodents (the vole *Microtus mogollonensis* and the mouse *Mus musculus*) and two primates (the macaque *Macaca mulatta* and the baboon *Papio hamadryas*). Lafuma et al. (8) identify the importance of the acceleration of the growth rate during the earliest stage of tooth development to achieving the increased molar complexity compared to mice. They also found that the mesiodistal and buccolingual dimensions of vole molar complexity and size have different biological etiologies. This is similar to patterns discovered for macaques (19), baboons (18), and shrews (not shown, 20) through the quantitative genetic analysis of adult tooth size variation. Image credit: The vole photograph of UTEP:Mamm:5422 is modified and used with permission from the University of Texas at El Paso’s webpage https://www.utep.edu/leb/pleistnm/taxamamm/arvicolinae.htm. The mouse, macaque, and baboon photographs are from the author.

This next step in realizing the true potential of evo-devo is hinted at in the studies of Lafuma et al. (8) and Sadier et al. (9). The patterns they identify in vole and bat dental development and evolution echo the genetic architectures of dental variation assessed through the quantitative genetic analysis of baboon (18), macaque (19), and shrew dental variation (20) (Fig. 1). These similarities in the signal of evolvability suggest that we are getting closer and closer to realizing an evo-devo that draws on and effectively intertwines the strengths of all of our resources: development, genetics, and the living and fossil evidence of evolution.
